# The Aryl Hydrocarbon Receptor Attenuates Acute Cigarette Smoke-Induced Airway Neutrophilia Independent of the Dioxin Response Element

**DOI:** 10.3389/fimmu.2021.630427

**Published:** 2021-02-15

**Authors:** Angela Rico de Souza, Hussein Traboulsi, Xinyu Wang, Jorg H. Fritz, David H. Eidelman, Carolyn J. Baglole

**Affiliations:** ^1^Research Institute of the McGill University Health Centre, Montreal, QC, Canada; ^2^Department of Medicine, McGill University, Montreal, QC, Canada; ^3^Department of Medicine, Western University, London, ON, Canada; ^4^Department of Microbiology and Immunology, McGill University, Montreal, QC, Canada; ^5^Department of Pathology, McGill University, Montreal, QC, Canada; ^6^Department of Pharmacology and Therapeutics, McGill University, Montreal, QC, Canada

**Keywords:** inflammation, neutrophil, AhR, FICZ, cigarette smoke, lungs

## Abstract

Cigarette smoke is a prevalent respiratory toxicant that remains a leading cause of death worldwide. Cigarette smoke induces inflammation in the lungs and airways that contributes to the development of diseases such as lung cancer and chronic obstructive pulmonary disease (COPD). Due to the presence of aryl hydrocarbon receptor (AhR) ligands in cigarette smoke, activation of the AhR has been implicated in driving this inflammatory response. However, we have previously shown that the AhR suppresses cigarette smoke-induced pulmonary inflammation, but the mechanism by which the AhR achieves its anti-inflammatory function is unknown. In this study, we use the AhR antagonist CH-223191 to inhibit AhR activity in mice. After an acute (3-day) cigarette smoke exposure, AhR inhibition was associated with significantly enhanced neutrophilia in the airways in response to cigarette smoke, mimicking the phenotype of AhR-deficient mice. We then used genetically-modified mouse strains which express an AhR that can bind ligand but either cannot translocate to the nucleus or bind its cognate response element, to show that these features of the AhR pathway are not required for the AhR to suppress pulmonary neutrophilia. Finally, using the non-toxic endogenous AhR ligand FICZ, we provide proof-of-concept that activation of pulmonary AhR attenuates smoke-induced inflammation. Collectively, these results support the importance of AhR activity in mediating its anti-inflammatory function in response to cigarette smoke. Further investigation of the precise mechanisms by which the AhR exerts is protective functions may lead to the development of therapeutic agents to treat people with chronic lung diseases that have an inflammatory etiology, but for which few therapeutic options exist.

## Introduction

Cigarette smoking is the foremost preventable cause of mortality worldwide, 80% of which is attributable to one of three diseases: lung cancer, cardiovascular disease and chronic obstructive pulmonary disease (COPD). Chronic inflammation plays a central role in the pathogenesis of these diseases. Cigarette smoke initiates an early and robust inflammatory response characterized by the increased production of cytokines, chemokines, and lipid mediators and the continued recruitment of innate and adaptive immune cells. Leukocytosis is the main respiratory and systemic immune alteration in smokers (1, 2) with the presence of neutrophils being a hallmark of this acute inflammatory response. Although inhaled corticosteroids (ICS) are a mainstay in the treatment for many inflammatory lung diseases, airway neutrophilia caused by smoking is largely resistant to all of the current medications used to manage diseases such as COPD, highlighting the need for new, and effective therapies to treat tobacco-related pathologies.

One cellular pathway that offers therapeutic potential against cigarette smoke-induced diseases involves the aryl hydrocarbon receptor (AhR). Work from our lab has shown that the AhR suppresses cigarette smoke-induced lung neutrophilia (3, 4). The AhR is a ubiquitous and evolutionarily-conserved receptor/transcription factor that is highly expressed in the lung (5). There is high constitutive AhR expression in lung epithelial/endothelial cells and fibroblasts (4, 6–9), compared to the relatively low expression in cells of hematopoietic origin [e.g., monocytes/macrophages (10)]. The AhR is well-known to mediate the toxic effects of environmental man-made contaminants such as 2,3,7,8-tetrachlorodibenzo-*p*-dioxin (TCDD/dioxin). In the absence of ligand, the AhR is cytoplasmic. After dioxin binds, the AhR translocates to the nucleus and forms a heterodimer with the AhR nuclear transporter (ARNT). This AhR•ARNT complex binds to DNA sequences termed the dioxin response element (DRE), initiating the transcription of genes that comprise the AhR gene battery, the prototypical of which are the Phase I cytochrome P450 (CYP) enzymes such as CYP1A1. This genomic pathway, involving nuclear AhR localization and DRE binding, mediates the toxic responses (e.g., cleft palate, hepatomegaly) to dioxin (11, 12). Other xenobiotics that are also AhR ligands and known to cause toxic outcomes includes benzo[*a*]pyrene (B[*a*]P), a polycyclic aromatic hydrocarbon that is produced from the combustion of organic material. Thus, B[*a*]P is a component of both air pollution and cigarette smoke and is an AhR agonist (13). Although B[*a*]P does not cause dioxin-like toxicity, it is a known human carcinogen. However, not all ligands which activate the classic, DRE-dependent AhR pathway cause toxicity. FICZ (6-formylindolo [3,2-b] carbazole) is an endogenous ligand and derivative of tryptophan that is produced in the skin after ultraviolet (UV) exposure (14, 15) FICZ binds the AhR with high-affinity (*K*_*d*_ of 0.07 nM for FICZ vs. 0.48 nM for dioxin) (14, 16). Although FICZ may exhibit toxicity at high doses (17), FICZ is largely non-toxic despite activation of the genomic AhR pathway. Moreover, FICZ may offer protection against numerous inflammation-associated diseases including psoriasis (18) and inflammatory bowel disease (IBD) (19). Whether activation of the AhR by FICZ protects against neutrophilia in the lungs in response to cigarette smoke is not known.

It is still poorly understood how different AhR agonists cause such a dichotomy in outcomes. While this may reflect the fact that dioxin for example is not metabolized (and therefore causes persistent AhR activation), it is also possible that there are additional biological pathways involving the AhR that are independent of its response to dioxin and work through a mechanism that does not involve translocation of the AhR to the nucleus or binding to the DRE. This non-canonical AhR pathway may underlie many of the physiological roles of the AhR, including regulation of cholesterol biosynthesis (20) and suppression of inflammation (21). Additional physiological functions of the AhR now includes liver development, endotoxin tolerance and resistance to infection (18, 22–24). Thus, we further sought to evaluate whether the ability of the AhR to control lung neutrophilia in response to cigarette smoke was independent of its nuclear localization and ability to bind the DRE.

To delineate the mechanism through which the AhR attenuates acute cigarette smoke-induced inflammation, we combined our preclinical model of cigarette smoke exposure with analysis in genetic AhR mouse models. Not only do our data confirm the importance of AhR activation in suppressing airway inflammation but we also show using novel lineages of mice incapable of nuclear AhR localization or DRE binding, that a novel AhR pathway confers protection against smoke-induced lung inflammation. Importantly, we also show that AhR activation by FICZ attenuates cigarette smoke-induced neutrophilia. Thus, these data confirm that the AhR is a critical immune-modulatory protein in the lung. It could also be that selectively activating the AhR (to bypass the detrimental effects associated with DRE binding) could alleviate pulmonary inflammation due to inhaled toxicants such as cigarette smoke, and thus represent a novel and viable therapeutic strategy for cigarette smoke-associated diseases.

## Materials and Methods

### Reagents

All chemicals were purchased from Sigma (St. Louis, MO) unless otherwise indicated. The AhR antagonist (25) 1-Methyl-N-[2-methyl-4-[2-(2-methylphenyl)diazenyl] phenyl-1H-pyrazole-5-carboxamide (CH-223191) and FICZ were from Tocris Bioscience (Minneapolis, MN).

### Animals

AhR-knockout (*Ahr*^−/−^) mice were bred and maintained as previously described (4). Male and female mice were used as indicated. Briefly, a breeding scheme of heterozygous *Ahr*^+/−^ to *Ahr*^−/−^ was used, rendering mice of the *Ahr*^+/−^ genotype as littermates. As *Ahr*^+/+^ or *Ahr*^+/−^ mice do not exhibit any difference in the ability to be activated by AhR ligands or cigarette smoke (3, 4), *Ahr*^+/−^ mice were used as controls throughout the study. Breeding stocks for *Ahr*^*dbd*/*dbd*^ and *Ahr*^*nls*/*nls*^ mice were kindly provided by Dr. Chris Bradfield (University of Wisconsin) and bred-in house. *Ahr*^*dbd*/*dbd*^ mice express the AhR, which can bind ligand and translocate to the nucleus, but is incapable of binding the DRE and are thus resistant to dioxin-induced toxicity (12). *Ahr*^*nls*/*nls*^ mice harbor a mutant AhR that is unable to translocate to the nucleus (11). As the DBD and NLS mutations were created in 129 ES cells carrying the lower affinity *Ahr*^*d*^ allele, additional controls also include B6.D2N-Ahr^d^/J mice, which harbor the low affinity *Ahr*^*d*^ allele on C57BL/6 background (26). All animal procedures were approved by the McGill University Animal Care Committee and were carried out in accordance with the Canadian Council on Animal Care Committee (Protocol Number: 5933).

### Ligand Administration

CH-223191 was reconstituted in DMSO and diluted in PBS prior to be intraperitoneouly (IP)-injected (50 μg per mouse). A single injection of CH-223191 or DMSO was given to mice on day 0 and 1 h before the first smoke exposure on days 1–3. FICZ was reconstituted in DMSO and diluted in PBS; 1 μg/mouse was administered IP or intranasal one day before beginning the first smoke and then 1 h before each smoke exposure on days 1–3.

### Cigarette Smoke Exposure

For experimentation, age-matched (8–17 weeks) *Ahr*^−/−^ mice and *Ahr*^+/−^ littermate controls as well as *Ahr*^*nls*/*nls*^, *Ahr*^*dbd*/*dbd*^, *AhR*^*dbd*/*B*6^, and B6.D2N-Ahr^d^/J mice were exposed to cigarette smoke as described using a SCIREQ^®^ InExpose Exposure System (SCIREQ, Montreal, QC) (4, 13) Briefly, research cigarettes (3R4F; University of Kentucky, Lexington, KY) were smoked in groups of 4 at 1 puff/cigarette/15 s for a total of 1 h. Mice received whole-body exposure of mainstream smoke diluted with air. The amount of smoke particulates was monitored using a MicroDust Pro (Casella CEL; Buffalo, NY) and maintained at a cumulative particulate density (CPD) of 300 g/L. Mice received two smoke exposures per day, 4 h apart, on days 1 and 2 and a single exposure on day 3. Exposure to cigarette smoke for 4 weeks was performed as we have previously described (27). At the same time, control mice were exposed to room air and manipulated in an identical fashion and 24 hours after the final exposure, mice were euthanized by exsanguination.

### Bronchoalveolar Lavage (BAL) and Tissue Harvest

Lungs were excised and PBS (0.5 ml) was injected twice to lavage the lungs. The BAL was centrifuged and the supernatant separated from the cells. The cells were resuspended in PBS, counted, mounted onto slides using a CytoSpin (Thermo Scientific; Waltham, MA) and stained with Three Step Stain (Thermo Scientific, Waltham, MA). Lung tissue was collected and either frozen immediately in liquid nitrogen and stored at −80°C for protein/western blot analysis or stored in RNAlater^®^ (Qiagen) for subsequent mRNA analysis.

### Western Blot

Lung tissue was homogenized in RIPA buffer supplemented with protease inhibitor cocktail (Roche; Indianapolis, IN). Total protein concentration was quantified using the bicinchoninic acid (BCA) assay (Thermo Scientific; Waltham, MA). Protein samples were mixed with reducing sample buffer (Sigma-Aldrich; St. Louis, MO) and heated at 95°C for 5 min. Total protein (10 μg) was resolved on a denaturing 10% SDS-polyacrylamide gel and transferred to a PVDF membrane. Membranes were blocked with 5% w/v non-fat milk prepared in PBS containing 0.1% Tween. The primary antibody was added to the membranes and incubated overnight at 4°C or 2 h at room temperature. These antibodies include CYP1A1 (1:700, Santa Cruz Biotechnology; Santa Cruz, CA), ICAM-1 (1:1,000, R&D Systems; Minneapolis, MN) and total actin (1:50,000, EMD Millipore; Billerica, MA). Membranes were washed in PBS/Tween 0.1% and incubated with the secondary antibodies anti-rabbit IgG-HRP (1:10,000, Santa Cruz Biotechnology; Santa Cruz, CA) or anti-mouse IgG-HRP (1:10,000, Life Technologies; Carlsbad, CA). Signals were detected by chemiluminescence imaging and analyzed using ImageLab (Bio-Rad Laboratories; Hercules, CA). Band intensities were normalized to the average intensity of DMSO air controls within each individual experiment.

### Detection of Cytokine Levels

BAL fluid collected as described above was stored at −80°C until used. BAL cytokine levels were evaluated using Luminex^®^ technology (Milliplex xMAP, Millipore, Billerica, MA).

### qRT-PCR

Total RNA was isolated from homogenized lungs using QIAzol Lysis Reagent and miRNeasy^®^ Mini Kit (Qiagen) according to the manufacturer's protocol. cDNA was generated from DNAse-treated RNA using iScript II Reverse Transcription Supermix (Bio-Rad, Mississauga, ON). Quantitative PCR was then performed by addition of 1 μl cDNA and 0.5 μM primers with SsoFast^TM^ EvaGreen^®^ (Bio-Rad). Data acquisition and analysis was performed on a CFX96 Touch^TM^ qPCR Detection System (Bio-Rad). Primers sequences are as published (4, 13). Gene expression was analyzed using the ΔΔCt method and results are presented as fold-change normalized to housekeeping gene (*Actb* or *Gapdh*). Data are expressed as fold-change relative to the average level of air-exposed controls.

### Generation of Bone Marrow Chimera (BMC) Mice

*Ahr*^+/−^ and *Ahr*^−/−^ mice were 6 weeks of age on the day of irradiation and 8 weeks of age for the CS exposures. CD45.2 recipient mice were treated with 2.5 mL of enrofloxacin (Baytril) per 250 ml of drinking water. After 3 days, mice were irradiated using X-RAD smart irradiator (9 Gy, 225 kVp, 13 mA, 526 s, whole body). The mice were then left for 16 hours, at which time bone marrow from CD45.1 donor mice were collected, resuspend in PBS, filtered and counted. The concentration was adjusted to 20 × 10^6^ cells/mL in order to deliver 4 × 10^6^ cells in 200 μl of PBS/mouse intravenous to the irradiated mouse. Mice were kept on antibiotics for 2 weeks, after which, chimeric mice were exposed to air or CS for 3 days and BAL cells analyzed. To verify the level of chimerism, bone marrow cells were isolated from AhR-KO^AhR^ and the control AhR-KO^ko^ mice following the last CS exposure. Bone marrow cells were stained with antibodies against C45.1 and CD45.2 followed by analysis via flow cytometry.

### Statistical Analysis

Results are reported as means ± S.E.M. Statistical differences between group-mean values were determined by 2-way ANOVA followed by the Tukey's multiple comparisons test. A *P* < 0.05 was considered significant.

## Results

### Inhibition of AhR Activity With CH-223191 Exacerbates Acute Cigarette Smoke-Induced Airway Neutrophilia

**Figure 1:**
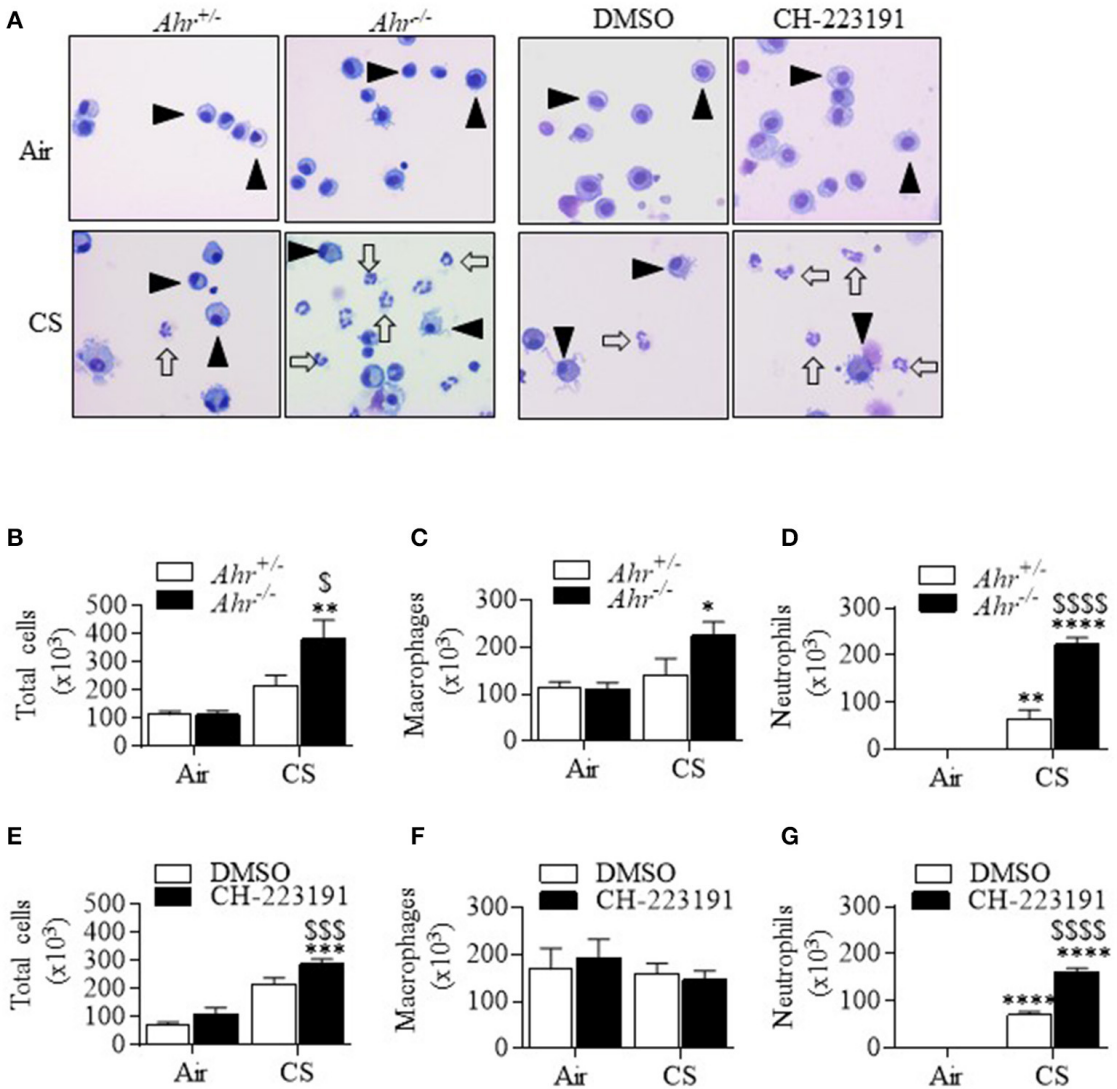
Inhibition of AhR activity by CH-223191 potentiates cigarette smoke (CS)-induced neutrophilia. **(A)** Note the presence of macrophages (*arrowheads*) as the predominant cell type in air-only mice. There were more neutrophils (*open arrows*) in the CS-exposed *Ahr*^−/−^ mice as well as *Ahr*^+/−^ mice pretreated with CH-223191 and exposed to smoke. Magnification = 20×. **(B)** There was a significant increase in total cells in *Ahr*^−/−^ mice exposed to smoke (**p < 0.01 CS compared to air; ^$^*p* < 0.05 smoke-exposed *Ahr*^−/−^ mice vs. CS-exposed *Ahr*^+/−^ mice). **(C)** There was a significant increase in macrophages in cigarette smoke-exposed *Ahr*^−/−^ mice compared to air (*p < 0.05). **(D)** There was a significant increase in neutrophils in smoke-exposed *Ahr*^+/−^ mice (**p < 0.01) as well as CS-exposed *Ahr*^−/−^ mice (****p < 0.0001). The increase in neutrophils in CS-exposed *Ahr*^−/−^ mice was significantly higher than in CS-exposed *Ahr*^+/−^ mice (^$$$$^*p* < 0.0001). **(E)** There was a significant increase in total cells in *Ahr*^+/−^ mice exposed to CH-223191 and CS (***p < 0.001 CS compared to air; ^$$$^*p* < 0.001 CS-exposed *AhR*^+/−^ mice receiving only DMSO vs. CH-223191. **(F)** There was no significant increase in macrophages. **(G)** Treatment with CH-223191 and exposure to CS significantly increased neutrophils in *Ahr*^+/−^ mice (****p < 0.0001). This increase was significantly higher than CS alone (^$$$$^*p* < 0.0001).

Cigarette smoke activates the AhR *in vitro* and *in vivo* to increase *Cyp1a1* expression (4, 13). This led us to wonder whether this AhR activation is necessary to suppresses smoke-induced neutrophilia. To address this, we administered the selective AhR antagonist CH-223191 (13, 25) IP to control (*Ahr*^+/−^) mice, and compared the effect of CH-223191 on pulmonary neutrophilia to that of smoke-exposed *Ahr*^−/−^ mice. Consistent with our previous publication (3), cigarette smoke exposure for 3 days significantly increased total cells as well as neutrophil numbers in *Ahr*^−/−^ and *Ahr*^+/−^ mice (Figure 1A—*open arrows*—and Figures 1B,D). However, the number of neutrophils was significantly higher in smoke-exposed *Ahr*^−/−^ mice (Figure 1D). There was an increase in macrophages only in smoke-exposed *Ahr*^−/−^ mice (Figure 1C). CH-223191 also significantly increased the number of total cells in smoke-exposed mice above those that received only cigarette smoke (Figures 1A,E). While there was no difference in macrophage numbers (Figure 1F), there was significantly more neutrophils in the airways of CH-223191-treated mice exposed to cigarette smoke compared to those which receive only DMSO (Figure 1G). Lymphocyte numbers did not change significantly after 3 days of cigarette smoke in any of the mice examined (data not shown). These results demonstrate that inhibition of AhR activity by CH-223191 mimics the phenotype of an AhR deficient mouse. Therefore, we conclude that AhR activation by cigarette smoke is an important factor in the ability of the AhR to suppress acute smoke-induced neutrophilia.

**Figure 2:**
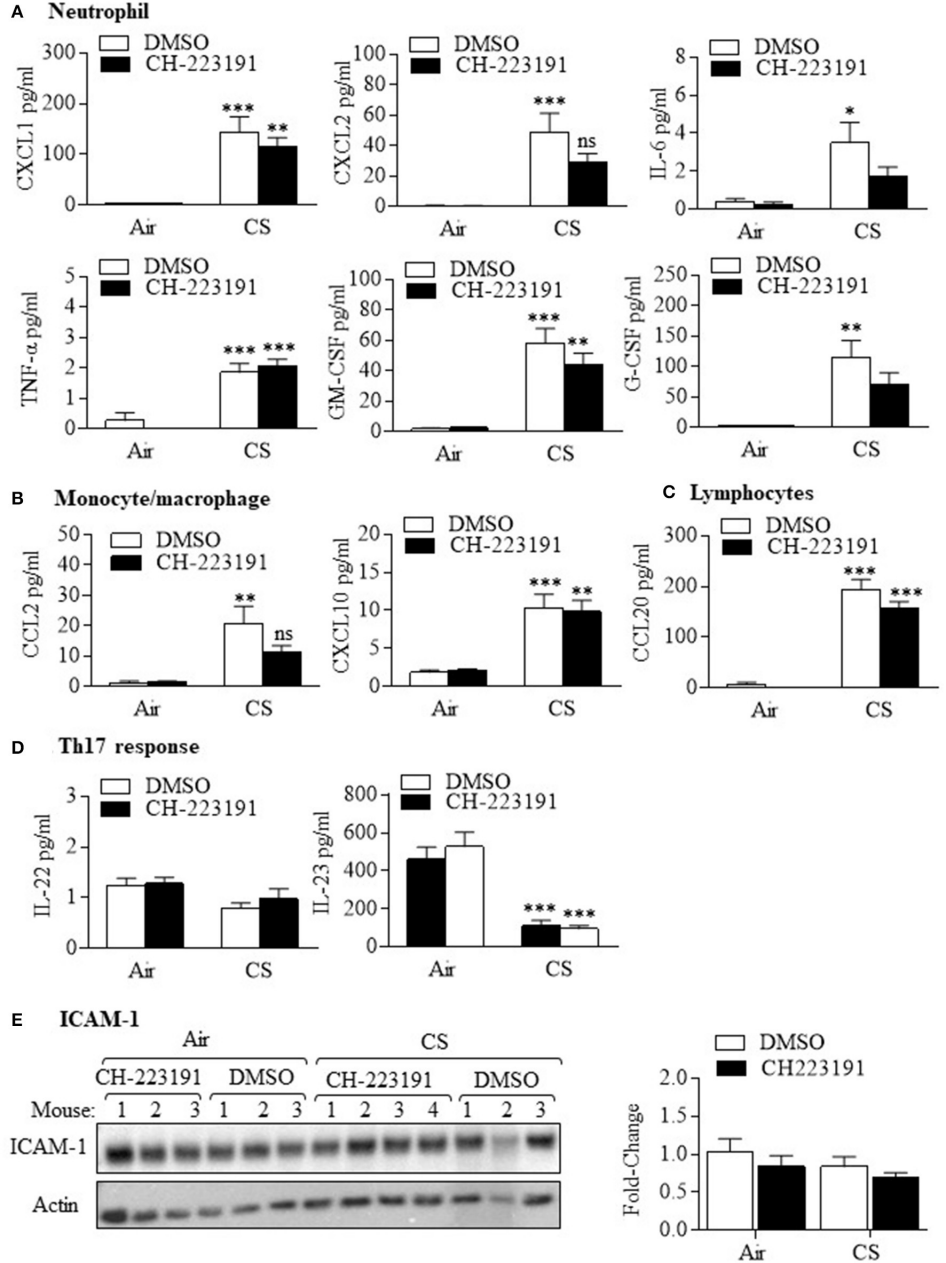
Cigarette smoke increases cytokine production independent of AhR activation. **(A)** Neutrophil—There was a significant increase in the neutrophil cytokines CXCL1, CXCL2, IL-6, TNF-α, GM-CSF, and G-CSF in response in mice exposed CS for 3 days that received only DMSO (*p < 0.05; **p < 0.01; ***p < 0.001 compared to air-exposed mice). There was no significant difference in the levels of these cytokines between smoke-exposed mice that received CH-223191 or DMSO. **(B)** Monocyte/macrophage—There was a significant increase in CCL2 and CXCL10 in *Ahr*^+/−^ mice exposed CS for 3 days that received only DMSO (**p < 0.01; ***p < 0.001 compared to air-exposed mice). There was no significant difference in the levels of these cytokines between CS-exposed mice that received DMSO compared to CH-223191. **(C)** Lymphocytes—There was a significant increase in CCL20 in *Ahr*^+/−^ mice exposed CS for 3 days that received CH-223191 or DMSO (***p < 0.001 compared to respective air-exposed mice). **(D)** Th17 response—There was no change in IL-22 levels with CS exposure. IL-23 levels decreased significantly with CS exposure in both DMSO and CH-223191-treated mice (***p < 0.001). Data represent two independent experiments (with at least three mice per group per experiment) and are shown as mean ± SEM. **(E)** ICAM-1- ICAM-1 was expressed in the lungs of all mice examined. Neither acute smoke exposure nor administration of CH-223191 had an effect on pulmonary ICAM-1. Blot shows results from individual mice (*n* = 3 per group) in a single experiment. Results are shown as means ± SEM.

Because of the predominant neutrophilic response after AhR inhibition with CH-223191 in smoke-exposed mice, we next examined the expression of cytokines in the BAL fluid that are strongly associated with neutrophil production and/or recruitment. The neutrophilic cytokines we examined included CXCL1 (GRO-α), CXCL2 (macrophage inflammatory protein 2-α [MIP2-α]), interleukin- 6 (IL-6), tumor necrosis factor-α (TNF-α), granulocyte-colony stimulating factor (G-CSF), and granulocyte-monocyte CSF (GM-CSF) (Figure 2A). While acute smoke exposure significantly increased the expression of these cytokines, there was no significant difference in the levels between mice treated with CH-223191 and exposed to cigarette smoke and those exposed to smoke and DMSO. There was also no difference in cytokines associated with monocyte/macrophage recruitment (Figure 2B) such as CCL2 (monocyte chemotatractant protein-1 [MCP-1]) and CCXL10 (interferon-γ-induced protein [IP-10]) between CH-223191 and DMSO mice also exposed to smoke. There was a also significant increase in the lymphocytic cytokine CCL20 in smoke-exposed mice but no difference between CH-223191 and DMSO-treated mice (Figure 2C). There was also no difference in the Th17-associated cytokines IL-22 and IL-23 (Figure 2D). Finally, pulmonary expression of the neutrophil adhesion molecule ICAM-1 in the lung was also not changed by smoke exposure or inhibition of AhR activity (Figure 2E). These data suggest that the regulation of neutrophilia in response to AhR inhibition by CH-223191 is not predominantly due differential control over chemotactic cytokines or increased expression of ICAM-1.

### The Attenuation of Cigarette Smoke-Induced Neutrophilia Is Independent of AhR Nuclear Localization or DRE Binding

**Figure 3:**
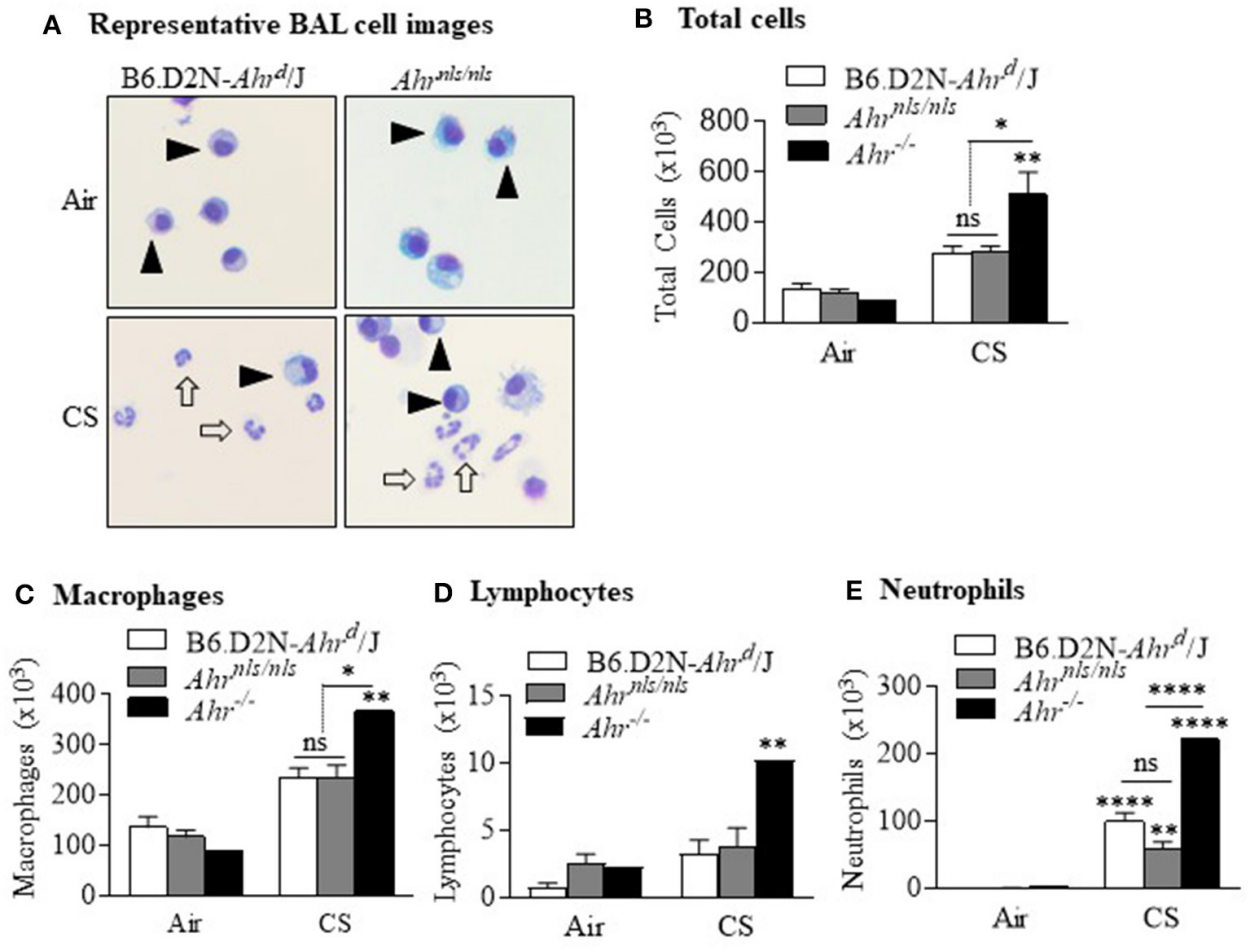
Suppression of acute smoke-induced neutrophilia does not require nuclear localization of the AhR. **(A)** Most cells within the BAL of air exposed B6.D2N-*Ahr*^*d*^/J and *Ahr*^*nls*/*nls*^ mice were macrophages (*arrowheads*). There was a noticeable increase in neutrophils after CS exposure (open arrows). Representative images are shown. **(B)** There was an increase in the total number of BAL cells after CS exposure (**p < 0.01). There was also a significant difference in total cells between the CS-exposed *Ahr*^−/−^ mice and both B6.D2N-*Ahr*^*d*^/J and *Ahr*^*nls*/*nls*^ mice (*p < 0.05) but not between B6.D2N-*Ahr*^*d*^/J and *Ahr*^*nls*/*nls*^ mice (ns). **(C)** There was an increase in the number of macrophages in CS-exposed *Ahr*^−/−^ mice. **(D)** There was no change in the number of lymphocytes. **(E)** There was a significant increase in the number of neutrophils after acute CS exposure (**p < 0.01; ****p < 0.0001 compared to respective air group). There was a stronger neutrophilic response in the *Ahr*^−/−^ mice compared to the CS-exposed B6.D2N-*Ahr*^*d*^/J and *Ahr*^*nls*/*nls*^ mice (****p < 0.0001). There was no difference in neutrophils between the CS-exposed B6.D2N-*Ahr*^*d*^/J and *Ahr*^*nls*/*nls*^ mice. Results are shown as means ± SEM (*n* = 5–12 male and female mice per group).

Our data that activation of the AhR was required to reduce smoke-induced neutrophilia led us to speculate whether the mechanism of this suppression involved translocation of AhR to the nucleus and binding to the DRE. To address this, we utilized two novel strains of mice-one with a mutation in the DRE (*Ahr*^*dbd*/*dbd*^) and another with a mutation in the nuclear localization sequence (*Ahr*^*nls*/*nls*^) (11, 12). As cigarette smoke causes nuclear translocation of the AhR (6), we first exposed *Ahr*^*nls*/*nls*^ mice to cigarette smoke for 3 days. For comparison, and in the same experiment we also included B6.D2N-*Ahr*^*d*^/J as well as *Ahr*^−/−^ mice. After a 3 day exposure to cigarette smoke, there was a noticeable increase in the presence of neutrophils in both the B6.D2N-*Ahr*^*d*^/J and *Ahr*^*nls*/*nls*^ strains (Figure 3A, *open arrows*). There was a trend toward an increase in total number of BAL cells in the B6.D2N-*Ahr*^*d*^/J and *Ahr*^*nls*/*nls*^ mice after smoke exposure that was not different between the strains (Figure 3B). Note that there was a significant increase in total cells in smoke-exposed *Ahr*^−/−^ mice (*black bar;* compared with Figure 1), an increase that was significantly higher compared to either B6.D2N-*Ahr*^*d*^/J or *Ahr*^*nls*/*nls*^ mice. There were significantly more macrophages (Figure 3C) and lymphocytes (Figure 3D) in the BAL of *Ahr*^−/−^ mice but not of the *Ahr*^*nls*/*nls*^ mice. There was a significant increase in neutrophils from smoke exposure in all strains examined, with the highest increase being in *Ahr*^−/−^ mice (Figure 3E). This neutrophilic response was significantly higher in *Ahr*^−/−^ mice but was not different between the smoke-exposed B6.D2N-*Ahr*^*d*^/J or *Ahr*^*nls*/*nls*^ mice. These data suggest that the suppression of smoke-induced neutrophilia by the AhR does not require its nuclear localization, and thus is likely independent of its transcriptional activity.

**Figure 4:**
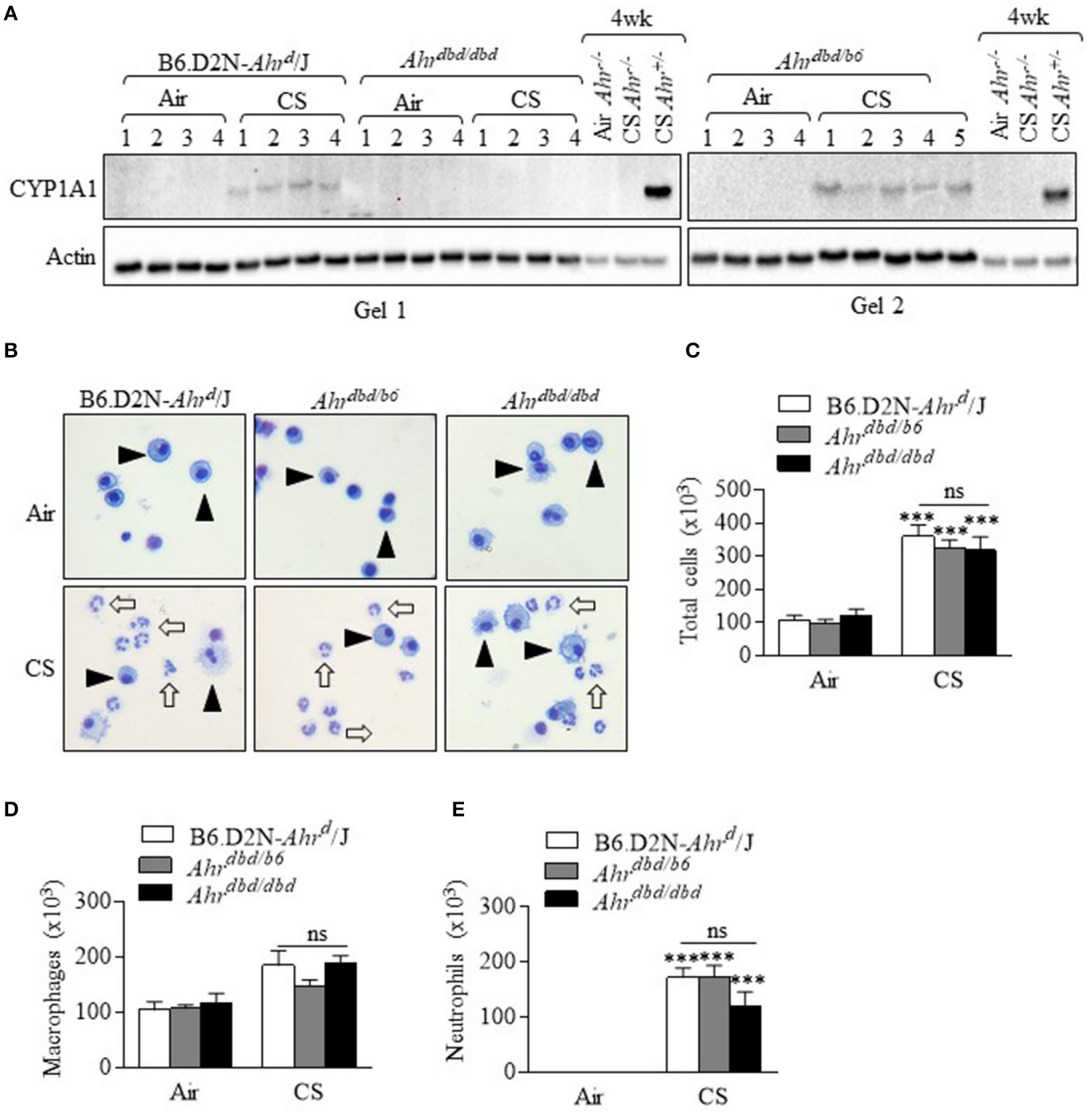
The AhR suppresses acute CS-induced neutrophilia independent of the DRE. **(A)** CS exposure for 3 days robustly increased CYP1A1 in the control mice (B6.D2N-*AhR*^*d*^/J- Gel 1 and Gel 2. There was no induction in CYP1A1 expression in *Ahr*^*dbd*/*dbd*^ mice (Gel 1). Additional controls included *Ahr*^−/−^ mice exposed to CS for 4 weeks (no CYP1A1) and strong CYP1A1 protein in *Ahr*^+/−^ mice. Numbers represent individual mice. **(B)** Macrophages were the predominant cell type in the BAL in the air exposed mice (*arrowheads*). Acute CS exposure noticeably increased the abundance of neutrophils (*open arrows*). Representative images are shown. **(C)** There was a significant increase in the total number of BAL cells from CS exposure (***p < 0.0001, CS vs. air-exposed mice). There was no significant difference (ns) in the total number of cells between the B6.D2N-*Ahr*^*d*^/J, *Ahr*^*dbd*/*b*6^, and *Ahr*^*dbd*/*dbd*^ mice. **(D)** There was no significant difference (ns) in the number of macrophages between the B6.D2N-*Ahr*^*d*^/J, *Ahr*^*dbd*/*b*6^ and *Ahr*^*dbd*/*dbd*^ mice. **(E)** CS exposure significantly increased the number of neutrophils in the BAL in each group (***p < 0.001 compared to air-exposed mice). There was no significant difference (ns) in the total number of neutrophils between the B6.D2N-*Ahr*^*d*^/J, *Ahr*^*dbd*/*b*6^ and *Ahr*^*dbd*/*dbd*^ mice exposed to CS. Results are shown as means ± SEM (*n* = 4–5 female mice per group).

**Figure 5:**
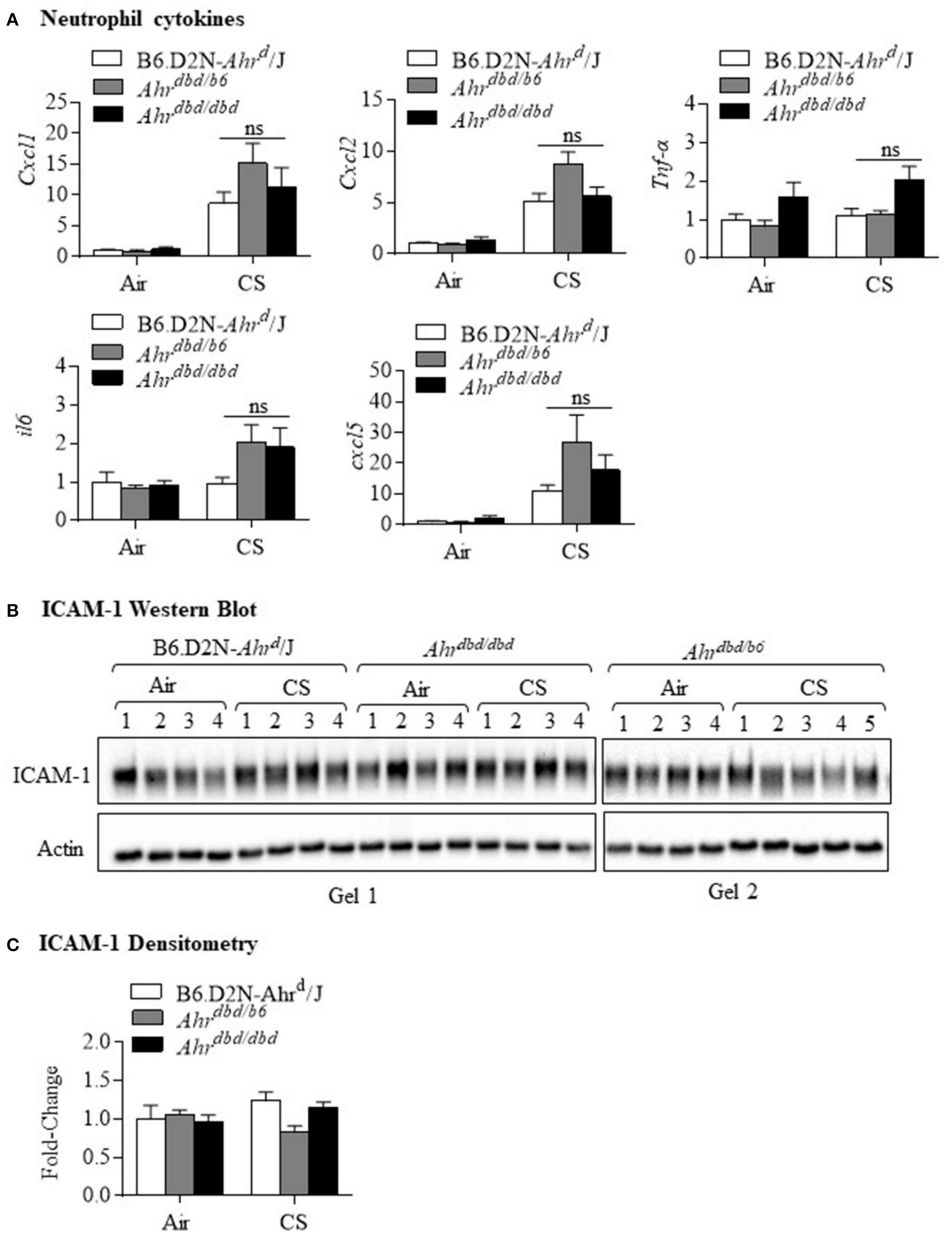
DRE-independent regulation of neutrophilic cytokines and adhesion molecule expression. B6.D2N-*Ahr*^*d*^/J, *Ahr*^*dbd*/*b*6^ and *Ahr*^*dbd*/*dbd*^ mice were exposed to CS for 3 days and cytokine mRNA assessed in whole lung homogenates. ICAM-1 levels were also evaluated by western blot. **(A)** Neutrophil cytokines—there was little difference in the induction of *Cxcl1, Cxcl2, Tnf-*α, *il6*, or *cxcl5* between the three groups of mice (ns = not significant). Results are shown as means ± SEM (*n* = 4–5 mice per group). **(B)** ICAM-1 Western blot—Pulmonary ICAM-1 levels were consistent between the B6.D2N-*Ahr*^*d*^/J, *Ahr*^*dbd*/*b*6^, and *Ahr*^*dbd*/*dbd*^ mice. There was little perceptible difference between the three groups in response to CS. **(C)** ICAM-1 Densitometry—There was no significant change in ICAM-1 protein expression after acute CS exposure. Results are shown as means ± SEM (*n* = 4–5 female mice per group).

Because our data support that nuclear localization of the AhR was not necessary for the attenuation of lung neutrophilia, it stood to reason that the response would also be DRE-independent despite the fact that smoke activates the AhR pathway which leads to DRE-dependent transcription. Although we have previously shown that cigarette smoke increases CYP1A1 expression in an AhR-dependent manner (4, 13), we have used the *Ahr*^*dbd*/*dbd*^ mice, together with *Ahr*^−/−^ mice, to confirm that the increase in pulmonary CYP1A1 expression by cigarette smoke exposure is DRE-dependent. Here, CYP1A1 was detected only in the control mice (B6.D2N-*Ahr*^*d*^/J, *Ahr*^*dbd*/*b*6^, and *Ahr*^+/−^ mice) exposed to cigarette smoke (Figure 4A). There was no detectable CYP1A1 expression after smoke exposure in *Ahr*^*dbd*/*dbd*^ or *Ahr*^−/−^ mice, a finding that is consistent with DRE-dependent control over CYP1A1 induction by classic AhR ligands (20). Next, we performed differential BAL cell counts after a 3-day cigarette smoke exposure regime in these mice. This analysis revealed that there was a noticeable and significant increase in the total number of cells in the control B6.D2N-*Ahr*^*d*^/J and *Ahr*^*dbd*/*b*6^ mice as well as the *Ahr*^*dbd*/*dbd*^ (Figures 4B,C). There was no statistical difference between the three groups of mice (Figure 4C). While macrophages were not significantly increased with this 3-day exposure regime (Figure 4D), there was a significant increase in lung neutrophilia in all three groups of mice (Figure 4E). However, there was no significant difference in neutrophils between *Ahr*^*dbd*/*dbd*^ mice and either the B6.D2N-*Ahr*^*d*^/J and *Ahr*^*dbd*/*b*6^ control mice. We also examined the mRNA expression for the cytokines *Cxcl1, Cxcl2, tnf-*α, *il6*, and *Cxcl5*. There was remarkably little difference—at the mRNA level—of these cytokines between the three groups of mice after exposure to smoke (Figure 5A). The level of ICAM-1 expression between *Ahr*^*dbd*/*dbd*^ mice and either the B6.D2N-*Ahr*^*d*^/J and *Ahr*^*dbd*/*b*6^ control mice was also not significantly different (Figures 5B,C).

**Figure 6:**
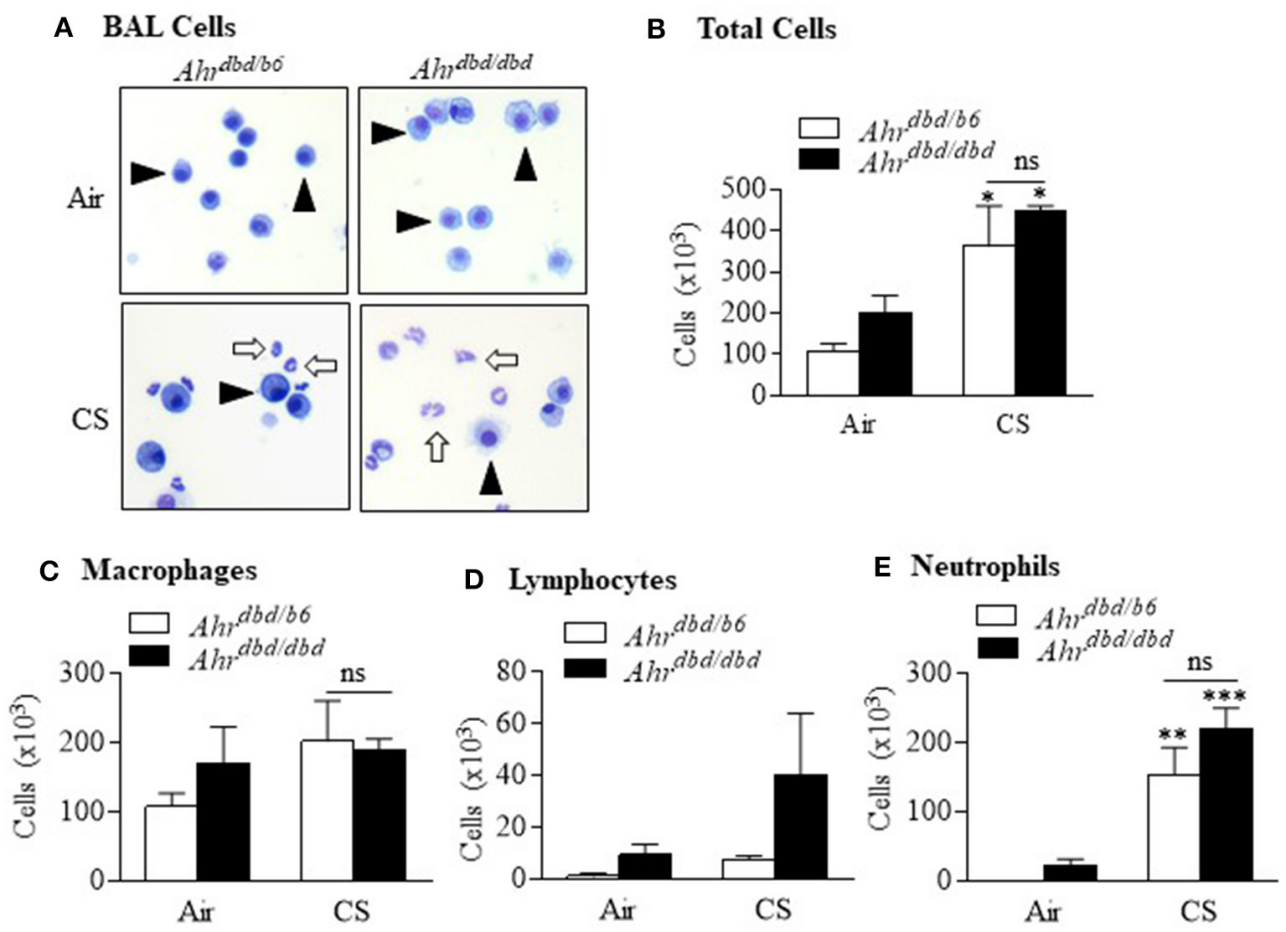
Suppression of sub-chronic cigarette smoke exposure is DRE-independent. *Ahr*^*dbd*/*b*6^ and *Ahr*^*dbd*/*dbd*^ mice were exposed to CS or room air for 2 weeks and differential cell counts in the BAL performed. **(A)** BAL cells—there was a noticeable increase in the presence of neutrophils (*open arrows*) after CS exposure (*arrowheads* indicate macrophages). **(B)** Total cells—there was a significant increase in the total number of cells in the BAL after CS exposure (*p < 0.05 compared to respective air exposed group). There was no significant difference in cells between CS-exposed *Ahr*^*dbd*/*b*6^ and *Ahr*^*dbd*/*dbd*^ mice (ns = not significant). **(C)** Macrophages—there was no difference in macrophage numbers between CS-exposed *Ahr*^*dbd*/*b*6^ and *Ahr*^*dbd*/*dbd*^ mice (ns = not significant). **(D)** Lymphocytes—while there was a trend toward more lymphocytes in CS-exposed *Ahr*^*dbd*/*dbd*^ mice, this increase did not reach statistical significance. **(E)** Neutrophils—there was a significant increase in the number of neutrophils in both the CS-exposed *Ahr*^*dbd*/*b*6^ (**p < 0.01) and *Ahr*^*dbd*/*dbd*^ mice (***p < 0.001) compared to room-air exposed mice. There was no significant difference (ns) in neutrophils between the CS-exposed *Ahr*^*dbd*/*b*6^ and *Ahr*^*dbd*/*dbd*^ mice. Results are shown as means ± SEM (*n* = 2–4 female mice per group).

We have previously published that the AhR can also reduce inflammation from prolonged smoke exposure (4), a finding that is relevant for people who smoke for years. Therefore, we also performed experiments and exposed *Ahr*^*dbd*/*b*6^ and *Ahr*^*dbd*/*dbd*^ mice to cigarette smoke daily for 2 weeks. These data further confirm that the ability of the AhR to maintain suppression for prolonged smoke exposures is also DRE-independent. Here, 2 weeks of daily cigarette smoke exposure in *Ahr*^*dbd*/*dbd*^ mice resulted in a level of neutrophilic inflammation that was not different from control mice (Figure 6). Thus, these data strongly suggest that the neutrophilic response to cigarette smoke is independent of the DRE.

### Suppression of Cigarette Smoke-Induced Neutrophilia Is Partially Due to AhR Expression in Cells of Hematopoietic Origin

**Figure 7:**
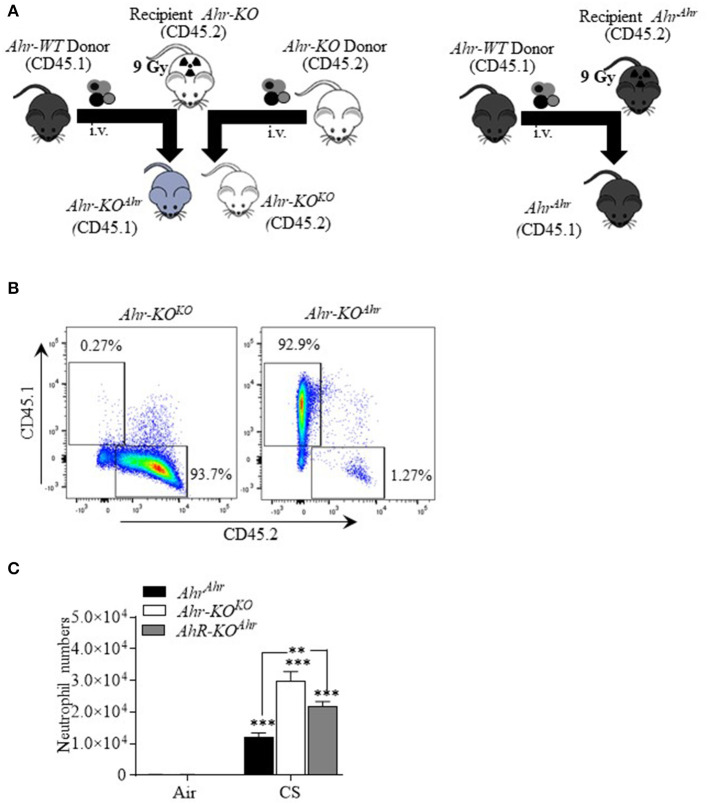
Suppression of CS-induced neutrophilia by hematopoietic and non-hematopoietic AhR expression. **(A)** Schematic of bone marrow chimeras: AhR-KO mice (recipient) were irradiated with 9 Gy and bone marrow cells transferred from *Ahr*-expressing (*Ahr*^*Ahr*^) mice to create chimeric mice in which the non-hematopoietic (structural) cells are KO but hematopoietic cells have AhR. Control mice (after irradiation of recipient) included *Ahr*^*Ahr*^ (all cells with AhR) and *Ahr-KO*^*KO*^ (all cells KO). **(B)** Flow cytometry of CD45.1 cells: Bone marrow cell subsets from *Ahr-KO*^*KO*^ (CD45.2) and BM chimeric mice *Ahr-KO*^*Ahr*^ (CD45.1) were labeled with antibodies for CD45.1 and CD45.2 and analyzed using flow cytometry. Data presented are representative dot plot. For the *Ahr-KO*^*Ahr*^ mice, BM cells were entirely donor derived (CD45.1) after reconstitution. **(C)** BAL neutrophils: acute (3 day) CS exposure significantly increased BAL neutrophils in *Ahr*^*Ahr*^ mice, *Ahr-KO*^*KO*^, and chimeric (*Ahr-KO*^*Ahr*^) mice compared to respective air-exposed mice (***p < 0.001). Neutrophils were significantly higher in *Ahr-KO*^*KO*^ (**p < 0.01) and *Ahr-KO*^*Ahr*^ mice (**p < 0.01) compared to *Ahr*^*Ahr*^ mice. There was some reduction in neutrophils in *Ahr-KO*^*Ahr*^ mice. *N* = 4–5 male mice per experimental condition.

The AhR is highly-expressed in structural cells within first-line defense organs such as the liver, skin, gut and lungs (7, 28). In cells of hematopoietic origin, such as monocytes/macrophages, AhR expression is low but increases with activation/polarization (29). Therefore, we wondered whether protection conferred against cigarette smoke is due to AhR activation in non-immune (i.e., structural) vs. hematopoietic cells. To address this, we generated bone marrow chimera (BMC) mice where structural cells are *Ahr-KO* but hematopoietic cells express AhR (*Ahr-KO*^*Ahr*^) (Figure 7A). The protocol used to generate the *Ahr-KO*^*Ahr*^ chimeric mice yielded almost complete reconstitution with donor bone marrow as indicated by the percentage of cells expressing CD45.1 (Figure 7B). To account for possible effects of radiation on the response to CS, and as additional controls, we also generated *Ahr-KO*^*KO*^ mice whereby AhR-KO mice were irradiated and received KO bone marrow cells (all cells without *Ahr*) as well as irradiated *Ahr*-expressing mice that received *Ahr* donor cells (*Ahr*^*ahr*;^ all cells express *Ahr*). There was a significant increase in neutrophilia in response to acute smoke exposure in all three groups of mice (Figure 7C). In *Ahr*^−/−^ mice reconstituted with KO bone marrow (*Ahr-KO*^*KO*^), the number of BAL neutrophils after smoke exposure was significantly higher compared to smoke-exposed *Ahr*^*Ahr*^ mice (Figure 7C—*white bar*); these data recapitulate the heightened neutrophilia in *Ahr*^−/−^ mice exposed to cigarette smoke for 3 days (3). In chimeric mice where structural cells are KO but bone marrow cells express AhR (*Ahr-KO*^*Ahr*^), there is significantly more BAL neutrophils than smoke-exposed *Ahr*^*Ahr*^ mice (Figure 7C). However, in the chimeric *Ahr-KO*^*Ahr*^ mice, there was significantly less neutrophilia compared to cigarette smoke-exposed *Ahr-KO*^*KO*^ mice (Figure 7C), highlighting the potential importance of AhR expression in hematopoietic cells in suppressing smoke-induced neutrophilia.

### AhR Activation by FICZ Attenuates Cigarette Smoke-Induced Neutrophilia

**Figure 8:**
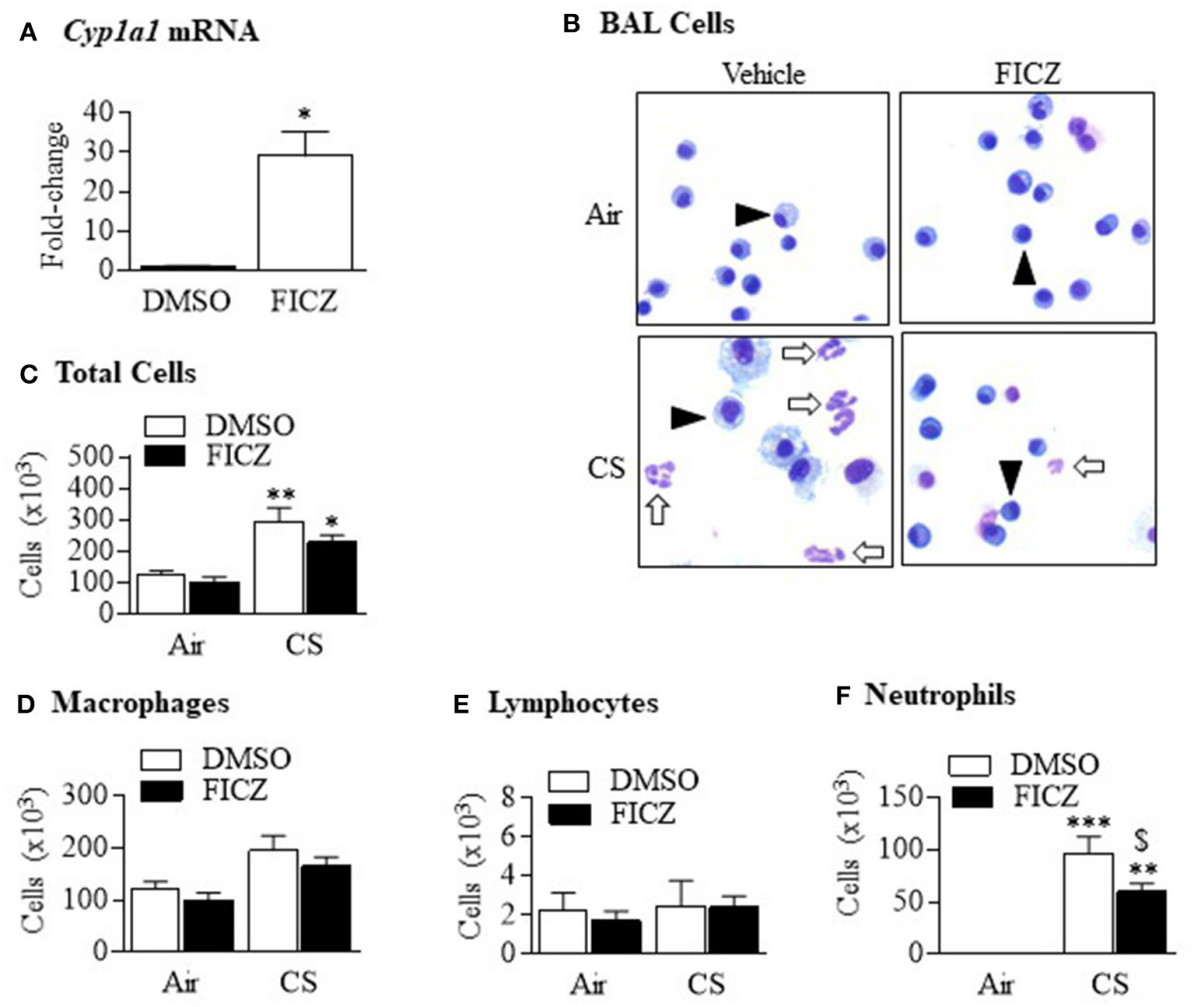
FICZ attenuates acute cigarette smoke-induced lung neutrophilia. *Ahr*^+/−^ mice were injected (i.p.) with FICZ 1 day before the beginning of the first smoke exposure and then 1 h before each subsequent exposure for 3 days. Differential cell counts on the BAL were performed. Whole lung homogenates were used to assess *Cyp1a1* mRNA expression by qRT-PCR. **(A)** There was a significant induction of *Cyp1a1* mRNA in the lungs of *Ahr*^+/−^ mice 6 h after receiving FICZ i.p. Results are expressed as means ± SEM (*n* = 2 mice per group). **(B)** There were neutrophils (arrows) detectable in the BAL of *Ahr*^+/−^ mice exposed to CS and receiving only DMSO (*open arrows*). There were noticeably fewer neutrophils in mice which received FICZ; macrophages are indicated by arrowheads. **(C)** There was a significant increase in the total number of cells after CS exposure (*p < 0.05; **p < 0.01). There was a trend toward fewer cells after FICZ. **(D)** There was no change in the number of macrophages after CS exposure or administration of FICZ. **(E)** Lymphocytes were not increased after 3 days exposure to CS or the levels modified by FICZ. **(F)** There was a significant induction in the number of neutrophils after exposure to CS (**p < 0.01; ***p < 0.0.001). Administration of FICZ significantly decreased the number of neutrophils caused by smoke exposure (^$^*p* < 0.0.05). Results are shown as means ± SEM (*n* = 7–8 male and female mice per group and are a compilation of two independent experiments).

**Figure 9:**
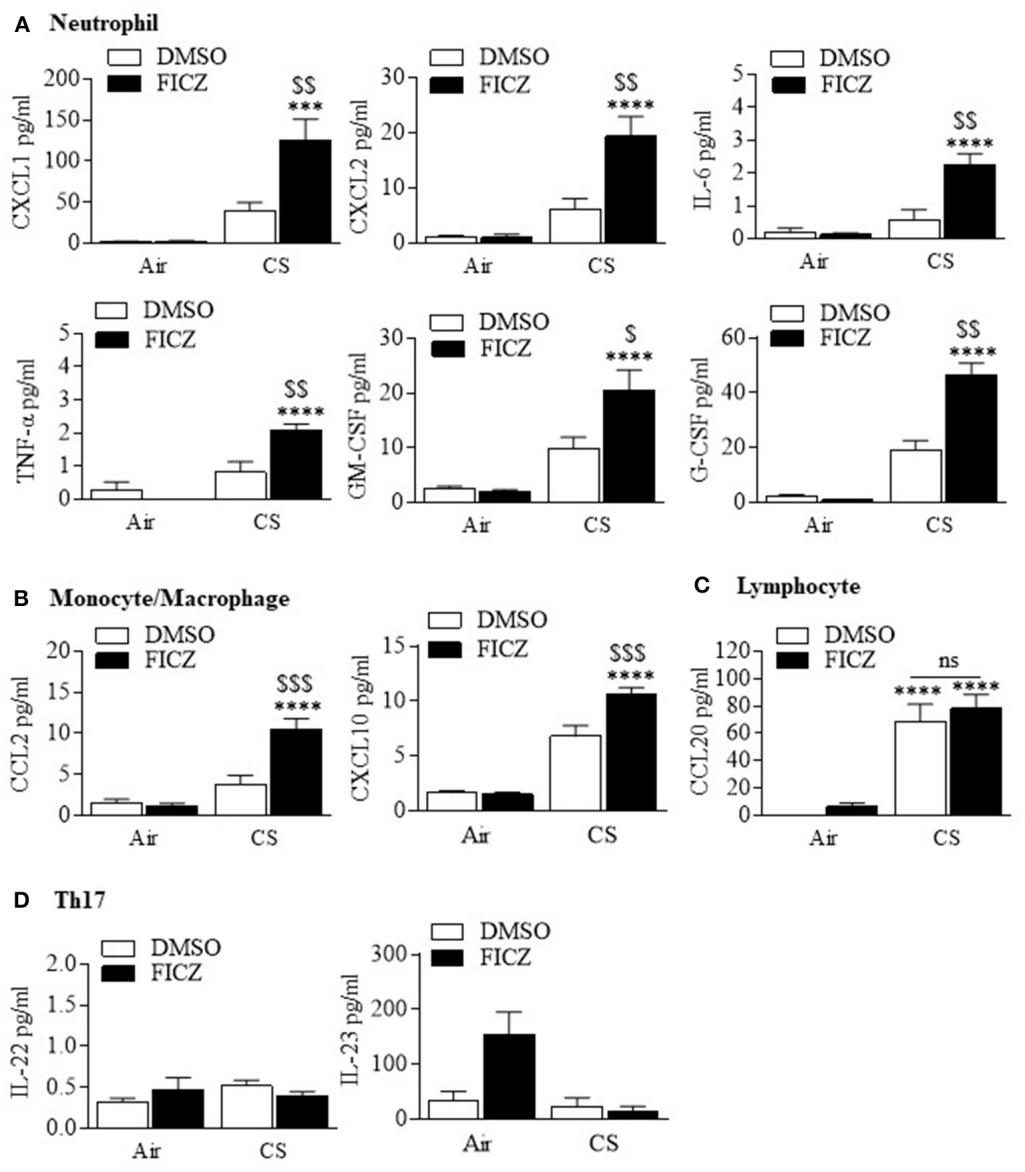
FICZ augments cigarette smoke induction of pulmonary cytokine levels. **(A)** Neutrophil—There was a significant increase in the levels of CXCL1, CXLC2, IL-6, TNF-α, GM-CSF, and G-CSF when mice were administered FICZ (***p < 0.001; ****p < 0.0001, compared to FICZ without CS exposure) and exposed to CS for 3 days compared to smoke exposure alone (^$^*p* < 0.05; ^$$^*p* < 0.01). **(B)** Monocyte/macrophage—There was a significant increase in CCL2 and CXCL10 when mice were administered FICZ (****p < 0.0001, compared to FICZ without CS exposure) and exposed to CS for 3 days compared to smoke exposure alone (^$$$^*p* < 0.01). **(C)** Lymphocyte- CS significantly increased CCL20 levels in the BAL (****p < 0.0001). FICZ had no effect on CCL20. **(D)** Th17—There was no change in IL-22 or IL-23 either with CS or FICZ administration. Results are shown as means ± SEM (*n* = 7–8 mice per group and are a compilation of two independent experiments).

Although studies have shown that FICZ attenuates the inflammatory response (18, 19), the ability of this endogenous AhR ligand to attenuate lung neutrophilia in response to cigarette smoke is not known. This is of particular importance to inhaled environmental toxicants such as smoke which induce an atypical inflammatory response that is resistant to current anti-inflammatory medications. Therefore, we first evaluated whether FICZ delivered IP to *Ahr*-expressing mice could attenuate acute smoke-induced neutrophilia. Administration of FICZ significantly increased *Cyp1a1* mRNA in the lung (Figure 8A), indicating that this administration regimen potently activates pulmonary AhR. Evaluation of the BAL (Figure 8B) revealed that cigarette smoke exposure increased the total number of cells (Figure 8C). While macrophages (Figure 8D) and lymphocytes (Figure 8E) were unchanged, cigarette smoke also increased the number of neutrophils to the lung (Figure 8F). However, administration of FICZ significantly decreased the number of neutrophils in response to acute cigarette smoke exposure (Figure 8F). Despite the fact that FICZ decreased the pulmonary neutrophilic response to smoke, there was actually an increase in the levels of numerous cytokines in the BAL involved in neutrophil recruitment, including CXCL1, CXCL2, IL-6, TNF-α, GM-CSF, and G-CSF (Figure 9). Cytokines associated with monocyte/macrophages such as CCL2 and CXCL10 (Figure 9B) were also increased by FICZ. Finally, cytokines associated lymphocyte recruitment (Figure 9C) and the Th17 response were unaffected by administration of FICZ (Figure 9D).

**Figure 10:**
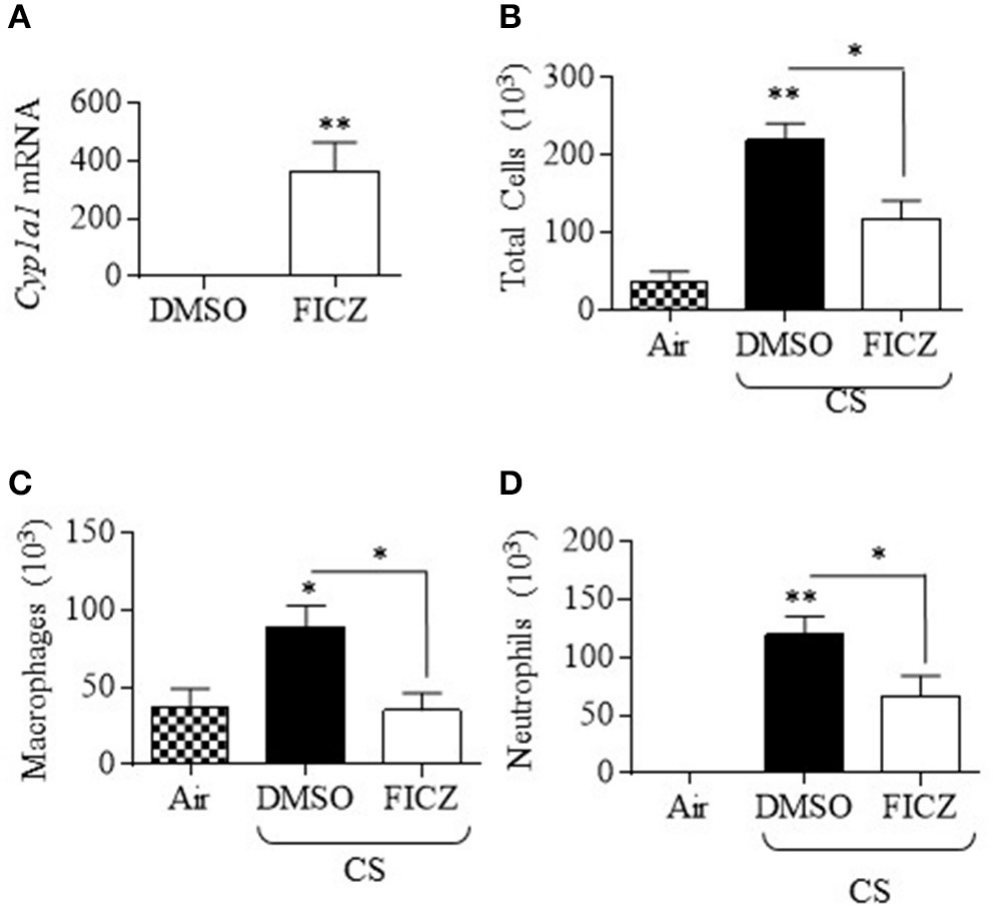
Pulmonary delivery of FICZ attenuates acute CS-induced lung neutrophilia. **(A)** Administration of FICZ via intranasal delivery (IN) significantly increased pulmonary *Cyp1a1* mRNA expression (*n* = 3 mice per group). This route of delivery also significantly decreased the number of total cells **(B)**, macrophages **(C)** and neutrophils **(D)** in the BAL in response to CS (*p < 0.05; **p < 0.01). Results are shown as means ± SEM (*n* = 2–4 male mice per group).

Because our data in the chimeric mice also support a contribution of AhR expression in non-hematopoietic cells in attenuating smoke-induced neutrophilia, and because the lungs would be the major target organ, lastly, we also performed experiments whereby we gave FICZ via intranasal delivery, as proof-of-concept that pulmonary administration of an AhR agonist is a viable delivery method for attenuation of this inflammatory response. This route of administration also yielded significant induction of pulmonary *Cyp1a1* mRNA (Figure 10A). There was a significant reduction in the total number of cells (Figure 10B) as well as macrophages (Figure 10C) and neutrophils (Figure 10D). We conclude that AhR activation an important factor in its ability to suppress smoke-induced neutrophilia.

## Discussion

Smoke-induced diseases such as COPD are characterized by abnormal airway inflammation, with heightened neutrophilia believed to play a role in disease pathogenesis. While the main treatment option for targeting inflammation is corticosteroids, these medications have no effect on neutrophilia caused by cigarette smoke (30, 31). Other approaches have included targeting specific mediators involved in the chemotaxis and accumulation of neutrophils (e.g., CXCL8, TNF-α) or downstream signal transduction pathways (e.g., p38), but these have either had little clinical benefit or been associated with significant side-effects (32, 33). Thus, the development of safe and effective anti-inflammatory therapies with disease-modifying effects for smokers with and without obstructive lung disease remains a priority. We speculated that the AhR is an essential protein in the homeostatic control of immunity in the lung. Herein, we show that AhR activation maintains a moderate level of neutrophilia, such that administration of FICZ attenuated—but did not eliminate—neutrophilia caused by smoke. This is important, given that strategies aimed at depleting neutrophils would render patients susceptible to respiratory infection. FICZ also protects against psoriasis (18), reduces inflammation in allergic asthma (34) and attenuates the severity of inflammatory bowel disease (IBD) (19). Furthermore, the AhR ligand from the lung, 2-(1′H-indole-s'-carbonyl)-thiazole-4-carboxylic acid methyl ester (ITE) (35) is also anti-inflammatory, suppressing the T-helper 17 (Th17) response in allergic rhinitis (36). Thus, defining the AhR as an important mechanism that underlies neutrophil migration to the lung may enable the development of safer approaches to treat chronic inflammatory conditions without the increased threat of infection.

We have previously reported enhanced inflammation, marked by increased accumulation of neutrophils in the airways, in *Ahr*-deficient mice after an acute exposure to cigarette smoke. This led us to theorize that activation of this receptor was important in its suppressive abilities. Overall, our data support the importance of AhR activation in preventing airway neutrophilia in response to cigarette smoke. When we used the AhR antagonist CH-223191 to inhibit AhR activation, the number of neutrophils significantly increased. This increase suggests a heightened inflammatory response, as neutrophils are usually the first cells recruited by damaged epithelial cells and tissue-resident macrophages. CH-223191 was initially shown to compete with dioxin, the most potent activator of the AhR, thereby preventing AhR translocation in mouse liver (25). We have shown that CH-223191 completely blocks smoke-induced AhR activation (13). As there is high constitutive AhR expression in lung structural cells (4, 6–9), it is possible that AhR activation within the lung itself by cigarette smoke is able to control the immune response. This is a possible scenario, as there is evidence that AhR expression in non-hematopoietic cells prevents neutrophilia in psoriasis and during influenza virus infection (18, 37). Our data also support that AhR in both hematopoietic and non-hematopoietic cells likely contributes to its overall ability to suppress pulmonary inflammation. One of the limitations of this study is that we did not determine the specific immune vs. lung structural cells whereby AhR expression is of most importance, although we speculate that it is AhR expression in multiple cells types that overall contributes to its homeostatic role in protecting the lung against toxicological insult. Nonetheless, our data support that activity of the AhR in the respiratory system is important for its ability to attenuate the neutrophilic response to cigarette smoke.

Neutrophil recruitment to the lung during injury or infection follows a cascade of tethering, rolling, adhesion, crawling, and transmigration (38), events that are mediated by chemically-diverse proteins including chemokines, cytokines, lipid mediators, and adhesion molecules. Many of these- including IL-8 (KC), IL-6, MIP-1α, CCL5 (RANTES), CXCL2 (MIP-2), and TNF-α- are increased by cigarette smoke exposure (4). It was therefore possible that the increase in neutrophil numbers upon inhibition of the AhR by CH-223191 would cause a corresponding change in neutrophil chemoattractants. However, there was no difference in any of the chemokines examined upon administration of CH-223191. This finding is also consistent with pulmonary neutrophilia by TCDD-induced AhR activation during influenza virus infection, where neither chemoattractants nor adhesion molecules were perturbed (37). We conclude that the regulation of neutrophilia by AhR activation cannot be accounted for solely based on differential regulation of chemotactic cytokines. This assumption is further strengthened by our data that administration of FICZ prior to and during the acute smoke exposure regime significantly attenuated the neutrophilic response despite significantly increasing neutrophilic cytokines. Many of these cytokines contain DREs in their regulatory region, including IL-6, IL-8, MCP-1, and TNF-α (39) and thus their induction by FICZ is not surprising. However, we cannot rule out the possibility that FICZ is increasing these cytokines indirectly via other regulatory sites or via interaction with other pathways. It should be noted that analysis of cytokines in our preclinical cigarette smoke model consistently shows increased levels of many pro-inflammatory mediators in the BAL in response to varying exposure times, both in this study and that which we previously published (3, 4). While there is variation in the absolute concentration of cytokines in the BAL (compare Figure 2 with Figure 9), and we cannot rule out issues related to reproducibility, this variation is likely a reflection of the fact that these represent biological samples from different experiments/mice, performed on different days and using separate assay kits. Overall, these results do suggest that alterations in cytokines do not account for the AhR regulation of neutrophilia.

It is unclear how the AhR suppresses neutrophilia. Our data suggest that changes in cytokines are unlikely to be how AhR prevents the exaggerated neutrophilic response. Other possibilities include increased expression of chemokine receptors on neutrophils. Splenic chemokine receptor expression is higher in *Ahr*^−/−^ mice (40), supporting the notion that higher receptor expression, rather than chemokines levels, may contribute to neutrophilia in *Ahr*^−/−^ mice upon smoke exposure. We also pursued the possibility that ICAM-1, an adhesion molecule crucial for the extravasation of neutrophils into tissues, was upregulated following cigarette smoke. However, our data suggest that changes in adhesion molecule levels is not an important contributor to the difference in neutrophil recruitment observed in our study. Another potential mechanism through which the AhR controls smoke-induced inflammation may be due to aberrant tissue repair mechanisms. Neutrophils secrete proteases and elastases that damage the lung epithelium, which recruits even more neutrophils. Thus, increased neutrophilia can self-propagate and promote further inflammation. A key player in tissue repair is IL-22, which is secreted by αβ CD4+ Th22 cells in adaptive immunity and γδ T-cell subset in innate immunity (41). However, levels of IL-22 did not change in response to AhR and/or smoke activation. Thus, it remains to be determined the mechanism through which AhR activation lessens smoke-induced lung neutrophilia and remains an active area of investigation.

Despite knowledge that the AhR mediates dioxin-induced toxicity, the physiological functions of the AhR remain largely unknown. Now, as our understanding of the AhR has increased, so too has insight into to its possible biological functions, including barrier integrity, immunity, reproduction, and vascular development (42). It is generally accepted that the deleterious health effects of dioxin arise from binding to the AhR, translocation to the nucleus and subsequent alterations in gene expression patterns due to DRE-dependent transcription. A key component to dioxin toxicity is its inability to be metabolized by enzymes strongly upregulated by AhR, including CYP1A1. This central dogma of the AhR has for decades precluded its utility as a pharmacological target and cast the AhR as merely a dictator of xenobiotic responses. However, the existence of alternative, non-genomic pathways for the AhR has come to light in recent years as an important contributor toward novel, non-xenobiotic functions of the AhR, involving cellular pathways such as Src (43), changes in intracellular calcium levels (44) and protein-protein interactions (45). Some non-genomic functions include the aggregation of platelets (46), control over cholesterol biosynthesis (20), T cell differentiation (47), cell migration (43) and regulation of protein expression (13, 48, 49). In this latter role, we have shown that the suppression of cigarette smoke-induced cyclooxygenase-2 (COX-2) expression by the AhR involved a DRE-independent pathway (13), leading us to further investigate whether the attenuation of acute neutrophilia by cigarette smoke was also DRE-independent. Using mice which express an AhR that is either incapable of nuclear translocation or DRE binding, we show that neither of these features of AhR signaling are necessary to attenuate the neutrophilic response, leading us to conclude that the inhibitory actions of the AhR against the inflammation-promoting effects of cigarette smoke occur largely through a non-genomic AhR pathway. Some additional non-transcriptional mechanisms could include interaction with the NF-κB family member RelB (3, 7) or control of function by the RNA-binding protein human antigen R (HuR) (13). While the precise DRE-independent mechanisms by which the AhR suppresses pulmonary inflammation remains to be determined, our data warrants further investigation into non-genomic AhR activation as a therapeutic strategy against inflammation associated with cigarette smoke exposure. It is also possible that the heightened neutrophilia associated with AhR deficiency is due to lack of metabolism of smoke components by CYP1A1 (as well as other Phase I and Phase II enzymes), as CYP1A1 is not expressed in *Ahr*-deficient mice. However, there is also no induction of CYP1A1 in response to smoke in the lungs of *Ahr*^*dbd*/*dbd*^ mice. Although we cannot conclude that differential expression of other proteins involved in metabolizing components of cigarette smoke account for the results obtained herein, we consider it unlikely that the results in this study can be accounted for by changes in CYP450 isoform expression. One of the limitations of our study is the focus on an acute model of cigarette smoke exposure, as this exposure duration does not allow for the development of airspace enlargement, a feature that is similar to the emphysema component of COPD and one that takes months (~4–6) of daily smoke exposure in mice to develop. However, this acute exposure model allowed us to interrogate mechanistically how the AhR suppresses smoke-induced neutrophilia, an immune cell type that is part of an inflammatory response whose dysregulation in susceptible smokers may contribute to lung disease (50).

In conclusion, we show that AhR activation can suppress inflammation in response to cigarette smoke. The discovery of the crucial connection between the AhR and its role in inflammation may aid the development of therapeutic agents to reduce the morbidity of chronic inflammatory respiratory diseases such as COPD. Together, these data warrant further investigation into non-canonical AhR activation in the lungs as a therapeutic strategy against toxicant-induced inflammation.

## Data Availability Statement

The raw data supporting the conclusions of this article will be made available by the authors, without undue reservation.

## Ethics Statement

All animal procedures were approved by the McGill University Animal Care Committee and were carried out in accordance with the Canadian Council on Animal Care Committee (Protocol Number: 5933).

## Author Contributions

AR, XW, and HT: data curation and analysis. CB: funding acquisition and supervision. AR and HT: methodology. AR, HT, JHF, and CB: project administration. AR, HT, DE, JHF, and CB: intellectual contributions and manuscript writing, review, and editing. All authors contributed to the article and approved the submitted version.

## Conflict of Interest

The authors declare that the research was conducted in the absence of any commercial or financial relationships that could be construed as a potential conflict of interest.
